# Single Amino Acid Polymorphisms in the *Fasciola hepatica* Carboxylesterase Type B Gene and Their Potential Role in Anthelmintic Resistance

**DOI:** 10.3390/pathogens12101255

**Published:** 2023-10-18

**Authors:** Estefan Miranda-Miranda, Raquel Cossío-Bayúgar, Lauro Trejo-Castro, Hugo Aguilar-Díaz

**Affiliations:** 1 Centro Nacional de Investigación Disciplinaria en Salud Animal, Instituto Nacional de Investigaciones Forestales Agrícolas y Pecuarias (INIFAP), Boulevard Cuauhnahuac No. 8534, Jiutepec 62550, Morelos, Mexico; aguilar.hugo@inifap.gob.mx; 2 Centro Nacional de Servicios de Constatación en Salud Animal SENASICA-SADER, Boulevard Cuauhnahuac No. 8534, Jiutepec 62550, Morelos, Mexico; lauro.trejo@senasica.gob.mx

**Keywords:** liver fluke, triclabendazole, amino acid substitution, binding pocket, ligand–protein docking, protein 3D modeling

## Abstract

The expression of the *Fasciola hepatica* carboxylesterase type B (CestB) gene is known to be induced upon exposure to the anthelmintic triclabendazole (TCBZ), leading to a substantial rise in enzyme-specific activity. Furthermore, the nucleotide sequence of the CestB gene displays variations that can potentially result in radical amino acid substitutions at the ligand binding site. These substitutions hold the potential to impact both the ligand–protein interaction and the catalytic properties of the enzyme. Thus, the objective of our study was to identify novel CestB polymorphisms in TCBZ-resistant parasites and field isolates obtained from a highly endemic region in Central Mexico. Additionally, we aimed to assess these amino acid polymorphisms using 3D modeling against the metabolically oxidized form of the anthelmintic TCBZSOX. Our goal was to observe the formation of TCBZSOX-specific binding pockets that might provide insights into the role of CestB in the mechanism of anthelmintic resistance. We identified polymorphisms in TCBZ-resistant parasites that exhibited three radical amino acid substitutions at positions 147, 215, and 263. These substitutions resulted in the formation of a TCBZSOX-affinity pocket with the potential to bind the anthelmintic drug. Furthermore, our 3D modeling analysis revealed that these amino acid substitutions also influenced the configuration of the CestB catalytic site, leading to alterations in the enzyme’s interaction with chromogenic carboxylic ester substrates and potentially affecting its catalytic properties. However, it is important to note that the TCBZSOX-binding pocket, while significant for drug binding, was located separate from the enzyme’s catalytic site, rendering enzymatic hydrolysis of TCBZSOX impossible. Nonetheless, the observed increased affinity for the anthelmintic may provide an explanation for a drug sequestration type of anthelmintic resistance. These findings lay the groundwork for the future development of a molecular diagnostic tool to identify anthelmintic resistance in *F. hepatica*.

## 1. Introduction

Zoonotic parasitic helminths present a substantial global threat to the health of both humans and livestock [1]. *Fasciola hepatica*, the causative agent of fascioliasis [2], stands out among these parasites. It affects millions of livestock, including bovines, ovines, equines, porcines, and camelids, and parasitizes millions of humans worldwide [3]. Fascioliasis is a significant livestock parasitosis in Mexico, ranking as the third most important and causing economic losses worth billions of dollars in the cattle and sheep industries [4]. Additionally, human fascioliasis is prevalent in Mexico, accounting for 13% of parasitic diseases in schoolchildren in the central Mexican state of Puebla [5], and serum antibodies against *F. hepatica* are present in 6% of the population in the central states, which are the most populous in Mexico [6]. Traditionally, anthelmintic drugs have been used to manage zoonotic fascioliasis by regularly administering them to humans, livestock, and other domestic animals. However, this approach is now facing challenges due to the emergence of anthelmintic resistance, a defensive mechanism employed by parasites against these drugs [1,7]. The development of anthelmintic resistance in *F. hepatica* is facilitated by the activity of xenobiotic metabolizing enzymes (XMEs) [8]. XMEs are present in various multicellular organisms and play a vital role in protecting against the toxicity of natural chemicals [9]. These enzymes possess the capability to deactivate xenobiotic compounds by modifying their hydrophilicity and accelerating their metabolism through a process known as biotransformation [1]. In parasitic helminths, including *F. hepatica*, a range of XMEs are involved, including cytochrome P450, monooxygenases, dehydrogenases, carboxylesterases, and others [9].

Carboxylesterase type B is an essential constituent of the xenobiotic metabolizing enzyme (XME) complex, which is present in a variety of organisms [10]. It possesses a diverse range of functions, including catalyzing hydrolytic reactions of carboxylic esters, phosphate esters, amides, thioesters, and other chemical compounds [11]. Carboxylesterase type B displays diverse substrate selectivity attributed to its spacious and adaptable binding pocket, which allows it to interact with various compounds, including pesticides [12]. However, the conformation of this binding pocket can be altered by single-nucleotide polymorphisms in the gene sequence, leading to amino acid substitutions that can affect the enzyme’s affinity for xenobiotics [13]. Carboxylesterase type B has been extensively studied for its role in xenobiotic sequestration, which provides resistance to pesticides in various organisms [14]. This phenomenon has been well documented in numerous examples, such as the hematophagous mosquito *Culex quinquefasciatus*, which demonstrates resistance to organophosphorus-based insecticides [15]; the red-spider mite *Tetranychus urticae*, which exhibits resistance to the acaricide spirodiclofen [16]; and the peach-potato aphid *Myzus persicae*, which shows resistance to pyrethroid and carbamate insecticides [17]. In instances of pesticide resistance, there is a significant increase in carboxylesterase type B expression levels and an enhanced interaction between the pesticide and the enzyme, even without the actual hydrolysis of the pesticide. This elevated expression of carboxylesterase type B effectively counteracts xenobiotics by binding to an equivalent amount of harmful chemicals, leading to the sequestration of xenobiotics [14].

Previous studies have shown that when exposed to triclabendazole (TCBZ), there is a significant increase in the expression of carboxylesterase type B (CestB) in *F. hepatica* [18]. TCBZ is commonly used as an anthelmintic to treat fascioliasis in animals and humans, and the emergence of TCBZ-resistant strains in *F. hepatica* is primarily associated with its widespread use [2,7]. The CestB gene in *F. hepatica* exhibits high levels of expression within the parasite. Its open reading frame encodes a protein consisting of 735 amino acids, resulting in a protein mass of approximately 85 kDa. This protein demonstrates the ability to hydrolyze a wide range of synthetic chromogenic carboxylic esters, encompassing various chemical structures [19]. Moreover, the analysis of *F. hepatica* CestB gene sequences has identified single-nucleotide polymorphisms (SNPs) that lead to amino acid substitutions at the ligand binding site. These substitutions can impact the enzyme’s affinity for the chromogenic substrate and potentially influence its catalytic properties [20].

The objective of this study was to investigate the impact of amino acid polymorphisms in the CestB gene of *F. hepatica*. Specifically, we focused on field isolates obtained from a highly endemic region of fascioliasis in Central Mexico, as well as reference strains of both TCBZ-susceptible and TCBZ-resistant parasites. Our main goal was to identify radical amino acid substitutions within the CestB sequence and examine their potential role in shaping the interaction between CestB and the anthelmintic drug TCBZ.

## 2. Materials and Methods

### 2.1. Animals and Parasite Strains 

All animal care and handling procedures conducted in this study were in strict adherence to the ethical guidelines set forth by our research institutions, following the Mexican norm NOM-062-ZOO-1999 and its technical specifications for animal production, care, and use. The reference strains of *F. hepatica* used in the anthelmintic bioassays were maintained at the Centro Nacional de Investigación Disciplinaria en Salud Animal (CENIDSAI/INIFAP, Mexico). These strains were registered in the NCBI BioSamples database and can be accessed using the identifiers SAMN16822856 for anthelmintic-susceptible *F. hepatica* and SAMN16822858 for TCBZ-resistant *F. hepatica* [21]. Additionally, the complete transcriptome sequences for these BioSamples were previously uploaded to GenBank and can be accessed at https://trace.ncbi.nlm.nih.gov/Traces/?view=run_browser&acc=SRR13076124&display=metadata, accessed on 1 September 2023.

### 2.2. Parasite Material

Between 10 and 20 adult *F. hepatica* parasites were collected at each location from the bile ducts of bovine and ovine hosts that were raised and subsequently sacrificed at official slaughterhouses in Central Mexico. To remove bile and any other adhering materials, the parasites were thoroughly rinsed with sterile phosphate saline solution (PBS) at pH 7.2 and a temperature of 37 °C. A section of the parasites devoid of eggs was carefully dissected and preserved in RNAlater^®^ (Thermo Fisher Waltham, MA, USA) for subsequent nucleic acid extraction, following a previously described protocol [22]. In addition, fecal samples were collected from areas where cattle grazed and processed in accordance with a previously reported method [23].

### 2.3. DNA Extraction

Genomic DNA was extracted from five adult parasites from each location; an egg-free fragment of each single adult parasite was used for DNA extraction using the standard phenol-chloroform procedure [24], and each DNA sample from each parasite’s fragment was processed independently for PCR and sequencing analysis. In addition, DNA isolation from fecal eggs was carried out on 2000 egg aliquots, as described in a previous study [23]. 

### 2.4. PCR Conditions

A set of primers (forward: FEx1CestB 5′-CGGGTCCAAGCAAGGATGAG-3′; reverse: REx1CestB 5′-CTCTCCTCCGACCATCAAATTC-3′) was designed based on the GenBank nucleotide sequences of CestB entries MT843326, MW655750, and OP537815. These primers were specifically designed to amplify exon one, spanning from nucleotide 99 to 1042, of the CestB gene [19]. The primers were generated using the Priming design tool algorithm at NCBI (https://www.ncbi.nlm.nih.gov/tools/primer-blast/, accessed on 1 September 2023). For polymerase chain reaction (PCR), a total volume of 20 μL was used, which contained 2 µL of Promega PCR master mix (10×), 0.5 µL of each primer at a 1 µM concentration, 1 µL of DNA (20 ng/µL), and 15 µL of H_2_O. The PCR amplifications were performed using the following cycling conditions: initial denaturation at 95 °C for 3 min, followed by 10 cycles of denaturation at 94 °C for 15 s, annealing at 65 °C for 30 s, and extension at 72 °C for 30 s. The thermocycler was programmed to decrease the annealing temperature by 1 °C per cycle. This was followed by 15 cycles of denaturation at 93 °C for 30 s, annealing at 60 °C for 30 s, and extension at 72 °C for 40 s. Finally, a final extension step was performed at 72 °C for 5 min. To ensure the reproducibility of the results, duplicate PCRs were carried out for each individual template DNA, and a negative control was included in all PCRs, which contained all components except for the DNA template.

### 2.5. Sanger Amplicon Sequencing

The amplicons were purified using the Wizard Gel and PCR Clean-Up system (Promega^®^ Madison, WI, USA) and subsequently sent for Sanger sequencing at the IBT-UNAM. The resulting DNA sequences were then translated into their respective amino acid sequences using the ORFinder tool available at NCBI (https://www.ncbi.nlm.nih.gov/orffinder/, accessed on 1 September 2023). To determine the presence of SNPs at positions 440, 643, and 788, as well as single amino acid polymorphisms (SAAPs) at positions 147, 215, and 263, a multiple sequence alignment was performed using the Clustal Omega online algorithm. This alignment encompassed both the CestB DNA and amino acid sequences obtained during the study (https://www.ebi.ac.uk/Tools/msa/clustalo/, accessed on 1 September 2023).

### 2.6. Protein Sequences and 3D Models

The amino acid sequences A0A8A1L7B4 and A0A4E0S0J7, together with their corresponding Alphafold 3D models in PDB files, were retrieved from the websites www.uniprot.org [25] and https://alphafold.com, accessed on 1 September 2023 [26,27]. Additionally, the complementary amino acid sequences of *F. hepatica* CestB, namely, MT843326, MW655750, OP537815, and THD28967.1, were obtained from www.ncbi.nlm.nih.gov, accessed on 1 September 2023. These sequences were then used to generate 3D models by submitting the FASTA amino acid sequences to https://robetta.bakerlab.org, accessed on 1 September 2023 [28]. The resulting models were further analyzed and annotated for their molecular properties.

### 2.7. Protein—Ligand Docking 3D Modeling

The 3D models of the oxidized anthelmintic ligand TCBZSOX and the chromogenic carboxylic ester ANA were obtained from https://pubchem.ncbi.nlm.nih.gov, accessed on 1 September 2023. To evaluate ligand docking and analyze the ligand binding site in three dimensions, we employed the CB-Dock2 online algorithm, which can be accessed at https://cadd.labshare.cn/cb-dock2/php/blinddock.php, accessed on 1 September 2023 [29,30]. The resultant 3D models of the protein–ligand complexes were downloaded as PDB files. For further modeling, visualization, and recording of the protein–ligand complexes, we utilized the Mol* online algorithm, which is accessible at https://molstar.org, accessed on 1 September 2023 [31].

## 3. Results

### 3.1. Parasites Samples

**Figure 1:**
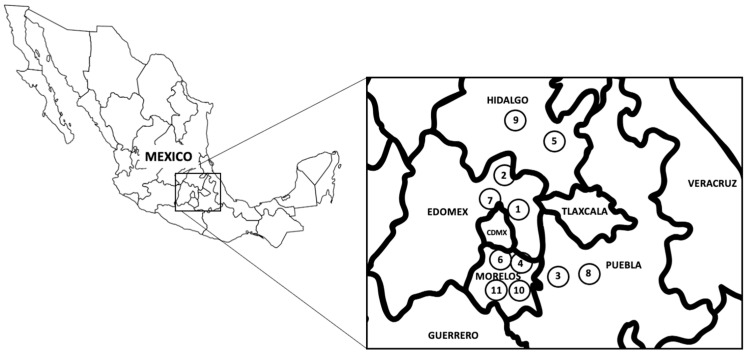
Location of *F. hepatica* field samples obtained from a highly endemic area that spans four states in Central Mexico. The coordinates of each location, along with the developmental stage of the samples, are comprehensively summarized in Table 1.

Samples of *F. hepatica* were collected from highly endemic areas in four central Mexican states as shown in Figure 1, and described previously [4,5,6,32]. Slaughterhouses in the study area sacrificed approximately 20 bovines and 15 ovines on a daily basis, with approximately four *F. hepatica*-parasitized livers detected in each slaughterhouse. Approximately 20 adult parasites were obtained from each location. The geographical coordinates and parasitic stage of each sample, as well as the different SNPs and SAAPs found in each CestB-sequenced sample, are compiled in Table 1. In addition, for the samples that exhibited polymorphisms during Sanger sequencing, the nucleotide at positions 440, 643, and 788 was represented as an R, indicating the possibility of either G or A. Furthermore, the SNPs resulting in amino acid substitutions at positions 147, 215, and 263 are also summarized in Table 1.

### 3.2. Protein 3D Modeling and Protein–Ligand Docking Analysis

**Figure 2:**
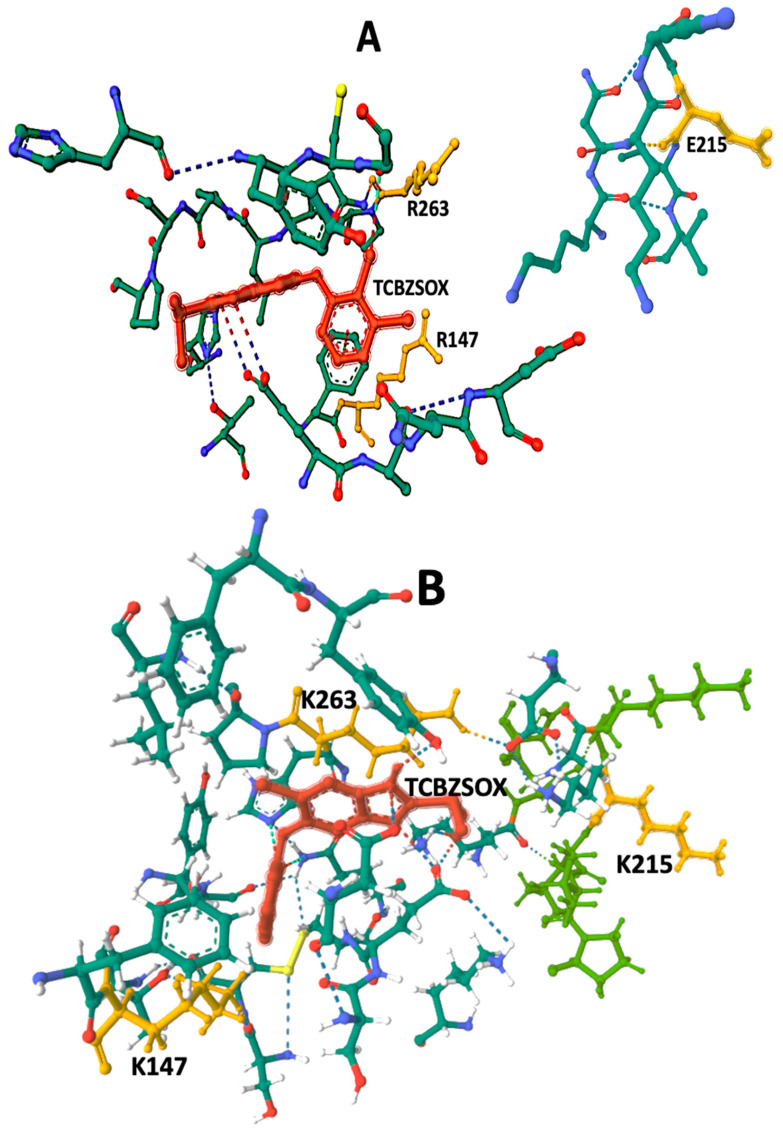
TCBZSOX-binding pocket in both TCBZ-susceptible and TCBZ-resistant strains of *F. hepatica*. Although TCBZSOX-binding pockets were identified in both anthelmintic-susceptible and anthelmintic-resistant parasites, marked differences were observed between the two polymorphisms. In the susceptible helminth polymorphism depicted in (**A**), the amino acids R147, E215, and R263 were located in separate domains, disconnected from each other, and distant from the position of the anthelmintic. Conversely, in the resistant strain shown in (**B**), the amino acid substitutions at K147, K215, and K263 led to the modification of the TCBZSOX-binding pocket, forming a compact domain that surrounds the anthelmintic. Notably, two of these amino acid substitutions, K147 and K263, highlighted in yellow, were found to directly interact with TCBZSOX. Amino acids at positions 147, 215 and 263 were highlighted in yellow, TCBZSOX was highlighted in red.

**Figure 3:**
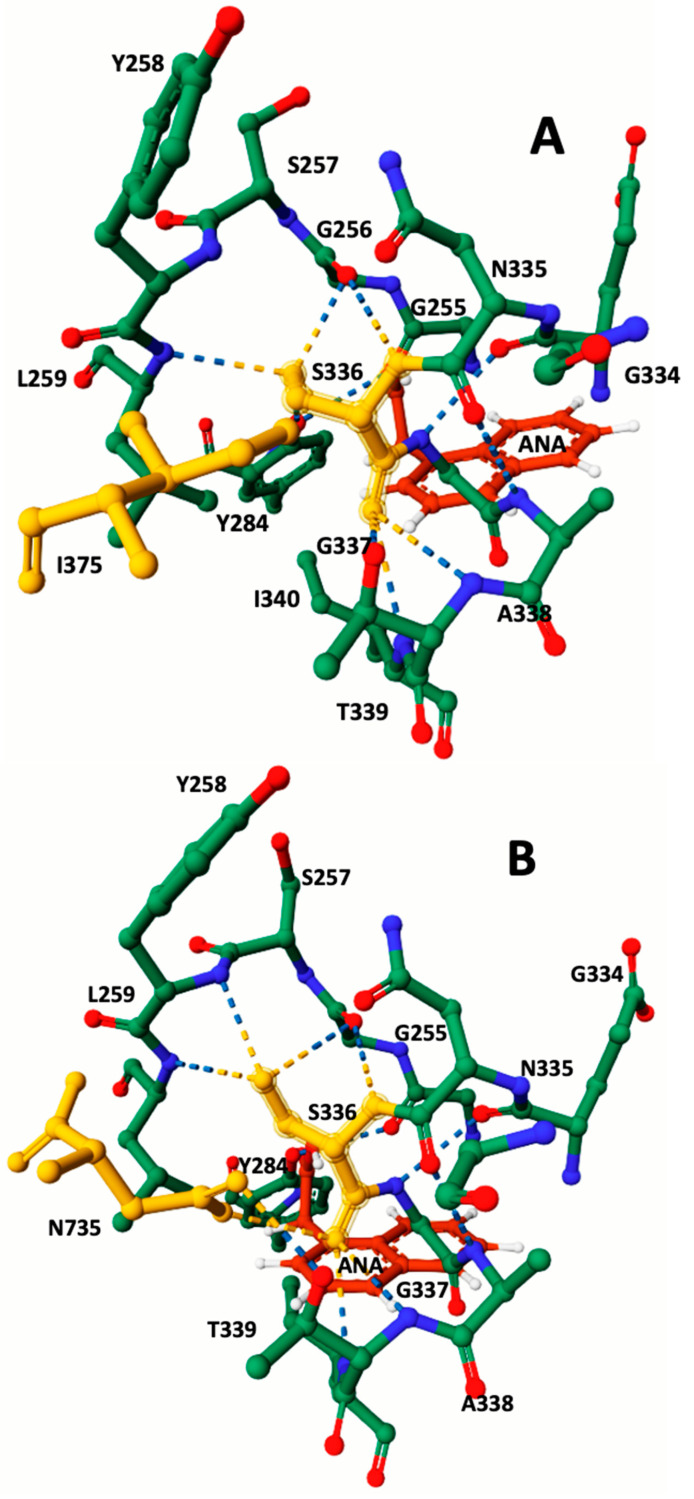
Detailed view of the CestB catalytic site featuring the 3D structures of the protein–ligand complexes. When analyzing the active site, the catalytic serine S336 highlighted in yellow was identified as a key component. Through protein–ligand docking analysis, different configurations were observed for the TCBZ-susceptible and TCBZ-resistant strains due to three amino acid substitutions. While these substitutions may not be visible in this figure and are not in close proximity to the catalytic serine 336, they still induce a structural reconfiguration at the core of the catalytic site. Notably, in the susceptible strain enzyme viewed in subfigure (**A**), the absence of N735 is replaced by I375 highlighted in yellow. In contrast, in the resistant strain viewed in subfigure (**B**), the presence of N753 highlighted in yellow, which is the last amino acid in the enzyme’s sequence, significantly brings it close to the catalytic S336, forming hydrogen bonds. The ANA ligand highlighted in red was employed to ascertain the position of the catalytic serine within the active site.

Amino acid sequences of *F. hepatica* carboxylesterase B from both susceptible and resistant strains were obtained from the GenBank and UniProt databases. Comprehensive information regarding the molecular properties of these sequences is presented in Table 2. To further analyze carboxylesterase B, Alphafold and Rosettafold 3D models were generated based on the amino acid sequences. These models were then employed in protein–ligand docking studies involving the metabolically oxidized anthelmintic TCBZSOX and the synthetic chromogenic carboxylesterase substrate ANA. The results of these docking experiments are summarized in Table 2. Moreover, Figure 2 and Figure 3 showcase detailed visual representations of the 3D models resulting from the docking process.

The CB-Dock2 algorithm was utilized to determine the amino acids constituting the TCBZSOX-binding site in the susceptible CestB enzyme isolated from the susceptible strain. The identified amino acids at this binding site included F146, R147, A199, S204, C206, G208, C225, T227, H262, R263, G265, L266, P267, P269, H637, and Y639. Interestingly, a different set of amino acids was observed at the TCBZSOX-binding pocket in the CestB amino acid sequence from the resistant strain, which comprised A281, I282, and S283, in addition to those present in the susceptible CestB amino acid polymorphism. These findings, along with the corresponding 3D models of the TCBZSOX-protein interactions, are summarized in Table 2 and depicted in Figure 2.

During the docking modeling analysis using the esterase chromogenic substrate ANA, the CB-Dock2 algorithm successfully identified the specific amino acids present at the catalytic site of the susceptible CestB. These amino acids were determined to be K210, N211, G214, E215, L216, V217, G256, Y258, L259, Y284, S336 and T339. Interestingly, in the resistant CestB, the amino acid I340 was replaced by N735, which was not observed in the susceptible polymorphism. These significant findings are succinctly summarized in Table 2, while the corresponding 3D models of the ANA-protein complex are represented in Figure 3.

## 4. Discussion

Anthelmintic resistance in parasites is a complex phenomenon involving genetic mechanisms that often require the collaboration of one or multiple genes to result in noticeable levels of resistance to anthelmintic treatment [1]. These genetic mechanisms can confer resistance through different phenotypes, including modifications in the target site of the anthelmintic [8] and/or increased enzymatic detoxification [9].

Our experimental findings revealed a significant disparity in the amino acid polymorphisms of CestB at positions 147, 215, and 263 between TCBZ-susceptible and TCBZ-resistant parasites, as presented in Table 1. These variations were also observed in the analysis of CestB field isolates, illustrating multiple combinations of the three amino acid substitutions (Table 1). These observations strongly suggest that the assortment of distinct amino acid polymorphisms found may be an essential environmental adaptation for the parasite.

To gain deeper insights into the underlying mechanisms driving the selection of the identified amino acid substitutions, we adopted a comprehensive approach encompassing both experimental and bioinformatics analyses. By leveraging this integrated methodology, we developed a 3D model aimed at elucidating the observed enzymatic disparities between the susceptible and resistant strains of the parasite. Notably, our study highlights that the specific combination of amino acid substitutions, namely, R147, E215, and R263, is exclusively present in TCBZ-susceptible parasites. Conversely, the combination of K147, K215, and K263 serves as a distinctive indication of TCBZ resistance in *F. hepatica*, as outlined in Table 1. Our study also uncovered an intriguing pattern of amino acid polymorphisms in the field isolates of the parasites, which have experienced consistent exposure to anthelmintic treatment within the livestock industry in Central Mexico [4]. Notably, these field isolates exhibited a high degree of heterozygosity at all amino acid substitutions, strongly indicating the occurrence of substantial CestB recombination. As *F. hepatica* is a hermaphroditic diploid organism capable of self-fertilization [21], each of the two genomes contains only one copy of CestB [20]. According to our experimental data, it is plausible that the two copies of CestB may encode different alleles, thereby leading to the diverse range of phenotypes observed in Table 1. This phenomenon can only be explained by sexual reproduction between two distinct parasites possessing different CestB alleles. Furthermore, our findings suggest that CestB polymorphisms may confer advantages to the parasite in overcoming immediate environmental challenges and potentially play a role in the development of anthelmintic resistance, as previously reported [19,22]. Additionally, our experimental data are consistent with the hypothesis of enzymatic detoxification, wherein the CestB amino acid polymorphisms may bind to, sequester, or enzymatically neutralize the anthelmintic compound, thus rendering it less effective [9].

In our investigation, we utilized TCBZ-CestB 3D docking modeling as part of our assessment. This approach involved the utilization of triclabendazole sulfoxide (TCBZSOX), the metabolically oxidized form of the anthelmintic triclabendazole. This biotransformation occurs within the liver of the host and results in an enhanced toxic effect of TCBZ on the parasites [2]. As a result, our 3D modeling analysis focused on evaluating ligand docking with TCBZSOX, specifically aiming to understand how the amino acid polymorphisms found in CestB influence the possible formation of a TCBZSOX-binding pocket.

The docking results reveal the presence of TCBZSOX-binding pockets in both strains’ enzymes, although there are noticeable differences. In the susceptible CestB strain, the amino acid substitutions R147, E215, and R263 are located in separate domains within the 3D model (Figure 2A), while in the resistant strain, the substitutions K147, K215, and K263 cause a change in the conformation of the binding pocket, forming a compact domain around TCBZSOX (Figure 2B). Moreover, in the resistant strain, the substitutions K147 and K263 directly interact with the anthelmintic, and T639 forms an ionic bond with TCBZSOX. None of these interactions were observed in the TCBZ-susceptible CestB model (Table 2).

Another notable distinction was the significant presence of hydrogen bonds between the amino acids in the TCBZSOX-binding pocket of the resistant strain and the anthelmintic. Specifically, amino acids such as D202, K210, H262, and P264 were observed to form hydrogen bonds in the resistant enzyme, as shown in Table 2 and Figure 2B. However, these specific interactions were absent in the enzyme of the susceptible parasite.

Ligand docking modeling with ANA yielded consistent results, indicating that the synthetic chromogenic substrate consistently positioned itself in close proximity to S336. This particular amino acid, known for its catalytic role in the hydrolysis of ANA into acetic acid and naphthol, was found to be crucial in the docking model [20]. This positioning of ANA in the model provided valuable insights into the location of the enzyme’s catalytic site while also highlighting the distinct conformations of constitutive amino acids between different parasite strains. Specifically, in the resistant strains, S336 displayed hydrogen bond interactions with G256, Y258, L259, T339, I340, and N735. Conversely, in the susceptible polymorphism, the catalytic serine formed hydrogen bonds with G256, Y258, L259, T339, and I340. These disparities in the active site of the enzyme align with previous findings and may help elucidate the observed increase in enzymatic specific activity in the resistant strain when compared to the susceptible strain [20]. To accurately determine the significance of substitutions in the development of the resistant phenotype, it will be essential to analyze field populations in Mexico as well as in other international locations. This comprehensive assessment will help establish the true importance of these changes in relation to triclabendazole resistance. If these substitutions are widely linked to resistance, it would open up the possibility of developing a specific molecular test for identification.

The docking model reveals an interesting finding: the TCBZ-binding pocket is situated far from the enzyme’s catalytic site, implying that the enzymatic hydrolysis of TCBZSOX is theoretically not possible. Nevertheless, the high level of expression that has been previously reported [19] aligns with the hypothesis of anthelmintic sequestration. Although the findings of this study are preliminary, they align with the concept that alterations in drug uptake, efflux, and metabolism are involved in the emergence of triclabendazole resistance in *F. hepatica*. Furthermore, the presence of amino acid polymorphisms in CestB suggests their potential involvement in this process. According to this hypothesis, excessive expression of the enzyme would generate enough free enzyme molecules to counteract the toxic effects of the anthelmintic, even without hydrolytic activity on the anthelmintic itself.

Our study offers compelling evidence supporting the idea that amino acid polymorphisms in CestB serve as a molecular indicator for TCBZ-resistant *F. hepatica*, making them potentially valuable in field isolates of the parasite. Additionally, our data strongly suggest that CestB likely plays a crucial role in liver fluke anthelmintic resistance by binding to TCBZ and potentially sequestering it.

## 5. Conclusions

Our findings support a hypothesis suggesting that one of the possible causes of anthelmintic resistance in the zoonotic parasitic trematode *F. hepatica* may involve amino acid substitutions occurring at the TCBZ-binding site of CestB, without involving the enzymatic hydrolytic degradation of the drug. Our study revealed a noteworthy level of polymorphism in *F. hepatica* CestB, with radical amino acid substitutions K147, K215, and K243 consistently detected in TCBZ-resistant parasites and field isolates. These substitutions effectively modify the TCBZ-binding pocket, allowing direct binding of the anthelmintic.

Furthermore, our investigation unveiled that these amino acid substitutions bring about a reconfiguration of the enzyme’s catalytic site, causing alterations in enzyme-specific activity against chromogenic carboxylic esters. This variance in enzymatic activity has been demonstrated in prior studies to possess statistical significance when comparing TCBZ-susceptible parasites with those displaying anthelmintic resistance [19].

In conclusion, our findings may have important implications for the development of a molecular test aimed at detecting anthelmintic resistance in *F. hepatica*. This could involve leveraging techniques such as qPCR, SNP analysis, and the measurement of carboxylesterase-specific activity using protein extracts from *F. hepatica*.

## Figures and Tables

**Table 1:** Summary of the nucleotide and amino acid polymorphisms observed in field isolates and reference strains of *F. hepatica* collected from a highly endemic area in Central Mexico. During automatic Sanger sequencing, positions 440, 643, and 788 exhibited heterogeneities, represented by the letter R, indicating that the nucleotide at these positions could be either G or A. Single amino acid substitutions (SAAPs) resulting from SNPs are depicted as heterozygosity R/K, E/K signifying an equal expression of arginine (R), glutamic acid (E) or lysine (K) at the respective position. Adult parasites were obtained specifically from official slaughterhouses, while eggs were obtained from fecal samples collected at grazing fields.

Sample	Location	Host Species	Parasite Stage	SNP440	SNP643	SNP788	SAAP147	SAAP215	SAAP263
1	19.5066° N, 98.8832° W	*Bos taurus*	ADULT PARASITES	R	A	R	R/K	K	R/K
2	19.8128° N, 99.0101° W	*Bos taurus*	EGGS	G	R	G	R	E/K	R
3	18.7649° N, 98.7147° W	*Bos taurus*	ADULT PARASITES	R	A	G	R/K	K	R
4	19.0095° N, 98.9983° W	*Ovis aries*	EGGS	G	G	G	R	E	R
5	20.1437° N, 98.6738° W	*Ovis aries*	EGGS	G	R	G	R	E/K	R
6	18.9848° N, 99.0931° W	*Bos taurus*	EGGS	G	R	G	R	E/K	R
7	19.6861° N, 98.8716° W	*Bos taurus*	ADULT PARASITES	G	R	G	R	E/K	R
8	20.9131° N, 98.4294° W	*Ovis aries*	EGGS	G	R	G	R	E/K	R
9	20.0905° N, 98.3691° W	*Ovis aries*	EGGS	R	A	G	R/K	K	R
10	18.6875° N, 99.1190° W	*Bos taurus*	EGGS	G	R	G	R	E/K	R
11	18.7718° N, 99.3534° W	*Bos taurus*	ADULT PARASITES	G	R	R	R	E/K	R/K
12	TCBZ-SUSCEPTIBLE	*Ovis aries*	ADULT PARASITES	G	G	G	R	E	R
13	TCBZ-RESISTANT	*Ovis aries*	ADULT PARASITES	A	A	A	K	K	K

**Table 2:** Amino acid composition of the TCBZSOX-binding pocket and catalytic site for both susceptible and resistant polymorphisms. To identify the specific amino acids comprising the affinity domain for the anthelmintic in TCBZ-sensitive and TCBZ-resistant *F. hepatica*, the CB-Dock2 algorithm employed TCBZSOX and ANA as ligands.

	TCBZ-Susceptible	TCBZ-Resistant
Amino acids in contact with TCBZSOX	F146, R147, A199, S204, C206, G208, C225, T227, H262, R263,G265, L266, P267, P269, H637, Y639	F146, K147, S204, A199, C206, G208, K210, K212, T227, H262, K263,P264, L266, A281, I282, S283, Y639
TCBZSOX-Binding Pocket Hydrogen Bonds	E198—TCBZSOX C640—TCBZSOX T144—H231 T639—H637 G265—R 263 D201—A199	D202—TCBZSOX K210—TCBZSOX H262—TCBZSOX P264—TCBZSOX T639—TCBZSOX D202—S204 G208—H262 G208—C206 K210—V217 K263—D623 G265—K263 P264—L266 C225—T227 G623—K212
TCBZSOX-Binding Pocket Ionic Bonds	F146—TCBZSOX	T639—TCBZSOX
Binding-Pocket Size Å^3^	1843	2233
Vina Score	−5.5	−8.3
AA Substitutions	147 R 215 E 263 R	147 K 215 K 263 K
Catalytic site AA Prediction	K210, N211, G214, E215, L216, V217, G256, Y258, L259, Y284, S336, T339, I340.	K210, N211, G214, K215, L216, V217, G256, Y258, L259, Y284, S336, T339, N735.

## Data Availability

Access to RNAseq data transcriptome from TCBZ-resistant and TCBZ-susceptible *F. hepatica* and supplemental information of the transcriptome are provided as links within the manuscript in the form of raw sequencing readings and tables that include the following: differential expression for each transcript in FPKM, GO, and KEGG numbers as well as GENBANK accession numbers for each sequence; details of generated raw reads; assembly and annotation information; overall transcriptomic annotation information such as mapping rate, number of known and unknown transcripts identified, splicing events and long noncoding RNA transcripts; and the annotated gene ontology divided according to the number of genes found as cellular components or fulfilling a biological process or molecular function.

## References

[1] Fissiha W., Kinde M.Z. (2021). Anthelmintic Resistance and Its Mechanism: A Review. Infect. Drug Resist..

[2] Brennan G.P., Fairweather I., Trudgett A., Hoey E., McCoy, McConville M., Meaney M., Robinson M., McFerran N., Ryan L. (2007). Understanding Triclabendazole Resistance. Exp. Mol. Pathol..

[3] Webb C.M., Cabada M.M. (2018). Recent Developments in the Epidemiology, Diagnosis, and Treatment of Fasciola Infection. Curr. Opin. Infect. Dis..

[4] Rodríguez-Vivas R.I., Grisi L., Pérez De León A.A., Silva Villela H., Torres-Acosta J.F.D.J., Fragoso Sánchez H., Romero Salas D., Rosario Cruz R., Saldierna F., García Carrasco D. (2017). Potential Economic Impact Assessment for Cattle Parasites in Mexico. Review. Rev. Mex. Cienc. Pecu..

[5] Zumaquero-Ríos J.L., Sarracent-Pérez J., Rojas-García R., Rojas-Rivero L., Martínez-Tovilla Y., Valero M.A., Mas-Coma S. (2013). Fascioliasis and Intestinal Parasitoses Affecting Schoolchildren in Atlixco, Puebla State, Mexico: Epidemiology and Treatment with Nitazoxanide. PLoS Negl. Trop. Dis..

[6] Sánchez Vega J.T., Morales Galicia A.E., Hernández López R., Navez Valle A., Morales Reyes E.G., Sánchez Aguilar D.I., Tapia Castor A.C., Coquis Téllez B., Hernández Covarrubias R.I. (2022). Panorama General de Las Helmintiasis Extraintestinales En México Durante Las Últimas Dos Décadas. Lux Méd..

[7] Fairweather I. (2011). Raising the Bar on Reporting ‘Triclabendazole Resistance’. Vet. Rec..

[8] Matoušková P., Vokřál I., Lamka J., Skálová L. (2016). The Role of Xenobiotic-Metabolizing Enzymes in Anthelmintic Deactivation and Resistance in Helminths. Trends Parasitol..

[9] Mordvinov V., Pakharukova M. (2022). Xenobiotic-Metabolizing Enzymes in Trematodes. Biomedicines.

[10] Wheelock C.E., Shan G., Ottea J. (2005). Overview of Carboxylesterases and Their Role in the Metabolism of Insecticides. J. Pestic. Sci..

[11] Hatfield M.J., Umans R.A., Hyatt J.L., Edwards C.C., Wierdl M., Tsurkan L., Taylor M.R., Potter P.M. (2016). Carboxylesterases: General Detoxifying Enzymes. Chem.-Biol. Interact..

[12] Bhatt P., Bhatt K., Huang Y., Lin Z., Chen S. (2020). Esterase Is a Powerful Tool for the Biodegradation of Pyrethroid Insecticides. Chemosphere.

[13] Newcomb R.D., Campbell P.M., Ollis D.L., Cheah E., Russell R.J., Oakeshott J.G. (1997). A Single Amino Acid Substitution Converts a Carboxylesterase to an Organophosphorus Hydrolase and Confers Insecticide Resistance on a Blowfly. Proc. Natl. Acad. Sci. USA.

[14] Cruse C., Moural T.W., Zhu F. (2023). Dynamic Roles of Insect Carboxyl/Cholinesterases in Chemical Adaptation. Insects.

[15] Hopkins D.H., Fraser N.J., Mabbitt P.D., Carr P.D., Oakeshott J.G., Jackson C.J. (2017). Structure of an Insecticide Sequestering Carboxylesterase from the Disease Vector *Culex quinquefasciatus:* What Makes an Enzyme a Good Insecticide Sponge?. Biochemistry.

[16] Wei P., Demaeght P., De Schutter K., Grigoraki L., Labropoulou V., Riga M., Vontas J., Nauen R., Dermauw W., Van Leeuwen T. (2020). Overexpression of an Alternative Allele of Carboxyl/Choline Esterase 4 (CCE04) of *Tetranychus urticae* Is Associated with High Levels of Resistance to the Keto-enol Acaricide Spirodiclofen. Pest Manag. Sci..

[17] Devonshire A.L., Field L.M., Foster S.P., Moores G.D., Williamson M.S., Blackman R.L. (1998). The Evolution of Insecticide Resistance in the Peach–Potato Aphid, Myzus Persicae. Phil. Trans. R. Soc. Lond. B.

[18] Scarcella S., Solana M.V., Fernandez V., Lamenza P., Ceballos L., Solana H. (2013). Increase of Gluthatione S-Transferase, Carboxyl Esterase and Carbonyl Reductase in Fasciola Hepatica Recovered from Triclabendazole Treated Sheep. Mol. Biochem. Parasitol..

[19] Pedroza-Gómez Y.J., Cossio-Bayugar R., Aguilar-Díaz H., Scarcella S., Reynaud E., Sanchez-Carbente M.D.R., Narváez-Padilla V., Miranda-Miranda E. (2021). Transcriptome-Based Identification of a Functional Fasciola Hepatica Carboxylesterase B. Pathogens.

[20] Miranda-Miranda E., Scarcella S., Reynaud E., Narváez-Padilla V., Neira G., Mera-y-Sierra R., Aguilar-Díaz H., Cossio-Bayugar R. (2022). A Single Nucleotide Polymorphism Translates into a Radical Amino Acid Substitution at the Ligand-Binding Site in Fasciola Hepatica Carboxylesterase B. Genes.

[21] Miranda-Miranda E., Cossio-Bayugar R., Aguilar-Díaz H., Narváez-Padilla V., Sachman-Ruíz B., Reynaud E. (2021). Transcriptome Assembly Dataset of Anthelmintic Response in Fasciola Hepatica. Data Brief.

[22] Scarcella S., Miranda-Miranda E., Cossío-Bayúgar R., Ceballos L., Fernandez V., Solana H. (2012). Increase of Carboxylesterase Activity in Fasciola Hepatica Recovered from Triclabendazole Treated Sheep. Mol. Biochem. Parasitol..

[23] Calvani N.E.D., Windsor P.A., Bush R.D., Šlapeta J. (2017). Scrambled Eggs: A Highly Sensitive Molecular Diagnostic Workflow for Fasciola Species Specific Detection from Faecal Samples. PLoS Negl. Trop. Dis..

[24] Sambrook J.F., Rusell D.W. (2001). Molecular Cloning: A Laboratory Manual.

[25] Bateman A., Martin M.-J., Orchard S., Magrane M., Agivetova R., Ahmad S., Alpi E., Bowler-Barnett E.H., Britto R., The UniProt Consortium (2021). UniProt: The Universal Protein Knowledgebase in 2021. Nucleic Acids Res..

[26] Jumper J., Evans R., Pritzel A., Green T., Figurnov M., Ronneberger O., Tunyasuvunakool K., Bates R., Žídek A., Potapenko A. (2021). Highly Accurate Protein Structure Prediction with AlphaFold. Nature.

[27] Varadi M., Anyango S., Deshpande M., Nair S., Natassia C., Yordanova G., Yuan D., Stroe O., Wood G., Laydon A. (2022). AlphaFold Protein Structure Database: Massively Expanding the Structural Coverage of Protein-Sequence Space with High-Accuracy Models. Nucleic Acids Res..

[28] Baek M., DiMaio F., Anishchenko I., Dauparas J., Ovchinnikov S., Lee G.R., Wang J., Cong Q., Kinch L.N., Schaeffer R.D. (2021). Accurate Prediction of Protein Structures and Interactions Using a Three-Track Neural Network. Science.

[29] Yang J., Roy A., Zhang Y. (2013). Protein–Ligand Binding Site Recognition Using Complementary Binding-Specific Substructure Comparison and Sequence Profile Alignment. Bioinformatics.

[30] Liu Y., Yang X., Gan J., Chen S., Xiao Z.-X., Cao Y. (2022). CB-Dock2: Improved Protein–Ligand Blind Docking by Integrating Cavity Detection, Docking and Homologous Template Fitting. Nucleic Acids Res..

[31] Sehnal D., Bittrich S., Deshpande M., Svobodová R., Berka K., Bazgier V., Velankar S., Burley S.K., Koča J., Rose A.S. (2021). Mol* Viewer: Modern Web App for 3D Visualization and Analysis of Large Biomolecular Structures. Nucleic Acids Res..

[32] Ibarra-Velarde F., Vera-Montenegro Y., Munguia-Xochihua J. (2011). Capítulo 9. Epidemiología de La Fasciolosis Animal y Humana. Epidemiología de Enfermedades Parasitarias en Animales Domésticos.

